# High-Performance Luminescent Solar Concentrators Based on Poly(Cyclohexylmethacrylate) (PCHMA) Films

**DOI:** 10.3390/polym12122898

**Published:** 2020-12-03

**Authors:** Francisco José Ostos, Giuseppe Iasilli, Marco Carlotti, Andrea Pucci

**Affiliations:** 1 Department of Physical Chemistry, Faculty of Chemistry, University of Seville, c/Prof. García González 1, 41012 Seville, Spain; 2 Dipartimento di Chimica e Chimica Industriale, Università di Pisa, via Moruzzi 13, 56124 Pisa, Italy; giuseppe.iasilli@gmail.com; 3 Center for MicroBioRobotics, Istituto Italiano di Tecnologia, viale Rinaldo Piaggio 34, 56025 Pontedera, Italy; marco.carlotti@iit.it

**Keywords:** poly(cyclohexylmethacrylate), poly(methylmethacrylate), Lumogen F Red 305, optical efficiency, luminescent solar concentrators, photovoltaics

## Abstract

In this study, we report on the use of poly(cyclohexylmethacrylate) (PCHMA) as an alternative to the commonly used poly(methylmethacrylate) (PMMA) for the design of efficient luminescent solar concentrators (LSCs). PCHMA was selected due to its less polar nature with respect to PMMA, a characteristic that was reported to be beneficial in promoting the fluorophore dispersibility in the matrix, thus maximizing the efficiency of LSCs also at high doping. In this sense, LSC thin films based on PCHMA and containing different contents of Lumogen F Red 305 (LR, 0.2–1.8 wt%) demonstrated optical efficiencies (η_opt_) comprising between 9.5% and 10.0%, i.e., about 0.5–1% higher than those collected from the LR/PMMA systems. The higher LR/polymer interactions occurred using the PCHMA matrix maximized the solar harvesting characteristics of the fluorophore and limited the influence of the adverse dissipative phenomena on the fluorophore quantum efficiency. These effects were also reflected by varying the LSC film thickness and reaching maximum η_opt_ of about 11.5% in the case of PCHMA films of about 30 µm.

## 1. Introduction

Climate change is one of the most relevant issues of our time. It is a shared opinion that the exponential increase in the energy demand has played (and sill plays) a major role in it, due to the vast use of fossil fuels and the related carbon dioxide emissions [1]. In this sense, renewable sources have become a powerful tool to reduce pollution. Among these, solar power is the cleanest and most abundant renewable energy source available and it can be converted into electrical or thermal energy through photochemical, photothermal, or photovoltaic processes. The latter consists in the direct conversion of sunlight into electricity and has been extensively investigated both in industry and academia for its simple scalability respect to power needs. Photovoltaic systems (PV) achieved tremendous progress in the past decades, leading to a 90% decrease in the energy price from 2007 to 2017 [2]. At the end of 2018, global PV capacity reached about 512 GW, of which about 180 GW (35%) were newly installed capacity [3].

However, because of their working principles, it is complex to integrate these solutions in the urban environment where the solar radiation is far from being optimally used. This is because the power produced by solar modules is directly proportional to the total power (i.e., the flux) of the incident light and thus the performances of these devices are drastically reduced when they operate in diffuse light or with a nonoptimal orientation [4]. As a result, currently, their use in cities is mostly limited to roof-top installations. A better and improved integration of solar power with the urban architecture, however, could bring an outstanding contribution toward the realization of greener and smarter cities of our future, where energy is produced directly where it is used, from renewable resources, and without requiring additional dedicated space [5].

**Figure 1:**
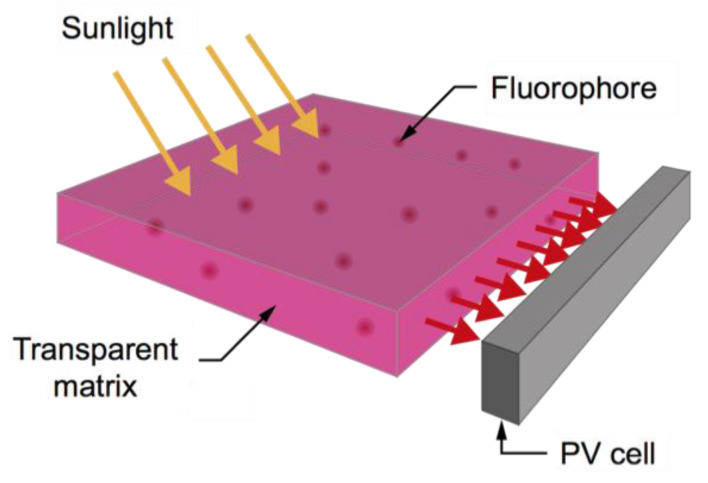
Working principle of the luminescent solar concentrators (LSC)/photovoltaic systems (PV) system.

Among possible systems for building-integrated PV, we can find luminescent solar concentrators (LSCs), optical systems intrinsically capable of trapping and concentrating light [6]. The concept of LSCs was first developed in the 1970s as an eco-friendly alternative to reduce the total cost of solar energy [7]. A typical LSC is made of a slab of a transparent matrix (the host), often a polymer, in which a fluorescent substance (the guest), often an organic dye with a high quantum yield, is dispersed (Figure 1) [8].

When the LSC/PV system is exposed to a radiation of a suitable wavelength, the latter is absorbed by the fluorophore, which then re-emits it at a longer wavelength by fluorescence. Because of the refractive index mismatch between the host and the air, this radiation remains trapped in the device by means of total internal reflection and it is emitted from the sides of the slab where it can be conveyed to a PV solar cell to produce electricity. Compared to the standard solar technology, the main advantages of these devices consist in their ability to work better than conventional designs in diffuse sunlight conditions (as in urban and domestic environments), they are transparent, and they have pleasing colors that can be used as functional architectural elements [9]. Several examples of working LSC prototypes can be found in the literature [10,11]. In addition, the technology of LSC can also be employed as spectral converter, shifting wavelengths of the solar spectrum to different ones that better match the regions of better efficiencies of solar cells [12].

However, light reabsorption and fluorescence quenching phenomena limit the PV performances in LSC [13]. To tackle this problem, much attention was given in the development of new fluorophores to improve the light concentration in LSCs cells. In particular, such compounds must possess specific characteristics to enhance the overall efficiency including broad absorption range, minimal self-absorption phenomena (i.e., large Stokes shift), high fluorescence quantum yields, long-term photo- and thermal stability (more than 20 years), and good solubility in the host matrix [14,15]. Commonly used fluorescent emitters include organic dyes [8,16,17,18,19], quantum dots [20], and lanthanide ions [21,22,23,24,25]. Among the wide variety of chromophores considered for LSCs, the perylenediimides [26,27]—and, in particular, the perylene-based fluorophores of the Lumogen series from BASF—represent the state-of-the-art in this field, thanks to the high quantum yields and long-term stability [28,29,30]. For instance, Lumogen F Red 305 (LR) is a fluorescent dye characterized by excellent photostability, efficient absorption in the visible light region, and high quantum efficiency with approximately 97% yield in poly(methylmethacrylate) (PMMA) [31], the most common host polymer for LSCs devices. In addition, among the proposed designs, the stacking of two or three layer configurations and the mixing of multiple fluorophores in the same bulk are also encountered in the literature to increase the overall LSC/PV power output [32,33].

Together with the characteristics of the dye, also the properties of the host matrix can affect the final performances of an LSC. The wide-spread use of PMMA for these applications, is due to the fact that it is commercially available, easy to process, and possess several optimal characteristics for LSC devices, such as a refractive index of about 1.50, a high glass-transition temperature (*T*_g_), good transparency, and good compatibility with most of organic fluorophores [34]. Nevertheless, it is also possible to overcome some of the limits of LSC devices by changing the nature of the polymeric host material [35]. Aimed to find accessible sustainable solutions, recent approaches suggested the use of biobased renewable resources as supporting matrices for LSC. L-poly(lactic acid) (L-PLA) [36], cellulose nanocrystals [37], fluorescent proteins [38,39], polyesters [40], and waterborne coatings [41] have been reported as sustainable alternatives to PMMA. In addition, poly(oxyalkylene)/siloxane organic–inorganic hybrid known as a ureasil and obtained from a sol–gel chemistry route have been recently employed as matrix for LSCs [42,43]. Over last years, improving the efficiency of PMMA as host matrix for LSCs have been a hot topic for many researchers. For example, Griffini et al. reported fluorinated polymers as matrix thin-film LSC devices with improved stability over time [44]. It was demonstrated that polymers with a more hydrophobic character than PMMA represent a viable alternative for LSCs [45,46]. Mansour et al. prepared a copolymer formed by styrene and methyl methacrylate (poly(STY-co-MMA)) doped with three different fluorophores, reporting an enhancement of the concentration efficiency for all tested copolymer/dye systems that the authors ascribed to the improved compatibility of the matrix with the apolar high quantum yield fluorophores [47].

**Figure 2:**
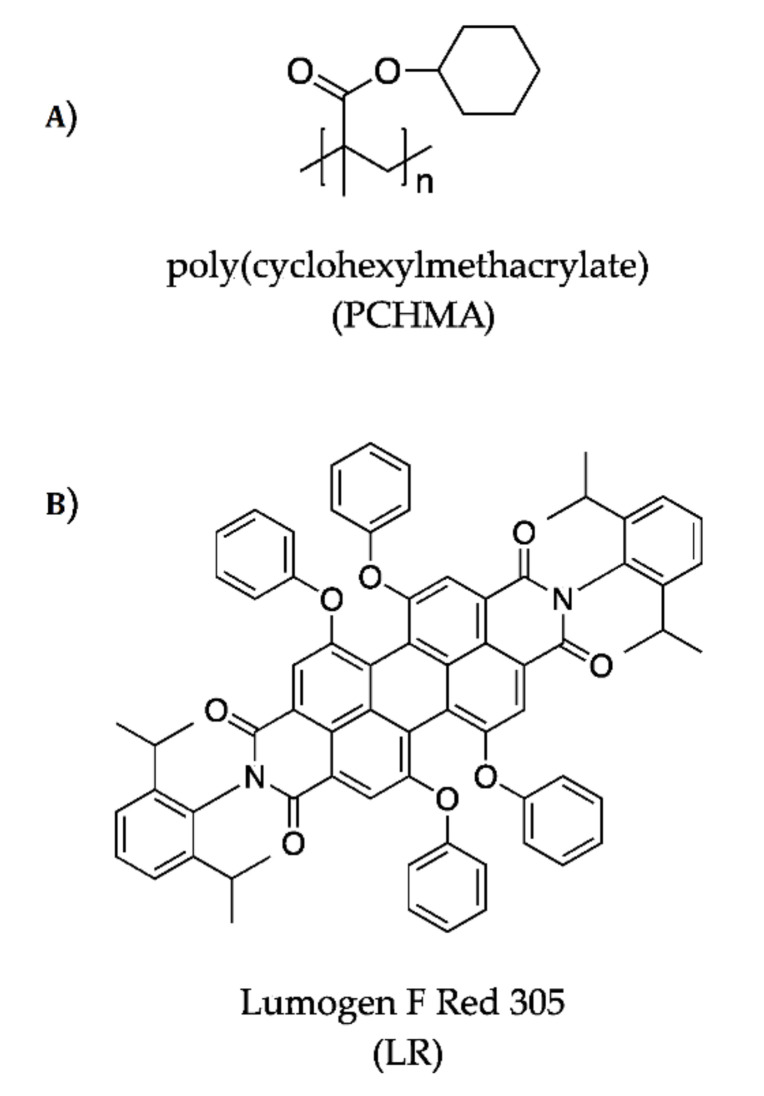
Chemical structures of (**A**) poly(cyclohexylmethacrylate) (PCHMA) and (**B**) Lumogen F Red 305 (LR).

On this account, we herein report on the performances of the commercially available poly(cyclohexylmethacrylate) (PCHMA) polymer matrix regarding the optical features and performances of thin-film LSCs. Notably, PCHMA (Figure 2A) was proposed in the literature as a more hydrophobic polymer matrix than PMMA that made it able to effectively disperse carbon fillers [48]. We selected LR (Figure 2B) as the state-of-the-art fluorophore for LSCs and we compared its optical efficiencies in PCHMA with those of standard PMMA-based thin-film solar collectors.

## 2. Materials and Methods

### 2.1. Materials

All the solvents were obtained from Sigma-Aldrich and used as received. Poly(cyclohexylmethacrylate) (PCHMA, M_w_ = 65,000 g mol^−1^, glass transition temperature (*T*_g_) = 104 °C) and poly(methylmethacrylate) (PMMA, M_w_ = 350,000 g mol^−1^, *T*_g_ = 105 °C) were purchased from Sigma-Aldrich (Sigma Aldrich, Milan, Italy) and used as received. Lumogen F Red 350 (LR) was kindly provided by BASF (BASF Colors & Effects Italy srl, Cesano Maderno (MB), Italy) and used without purification. Optically clear glass slides were cleaned by pouring them in 6 M HCl for 12 h, rinsing with water, acetone, and 2-propanol, and then drying for 8 h at 120 °C.

### 2.2. Preparation of Lumogen F Red 305/Polymer Films for Optical Studies

Different Lumogen F Red 305/polymer thin films were prepared by drop casting, i.e., pouring about 1.4 mL chloroform (CHCl_3_) solution containing about 60, 90, or 120 mg of polymer (i.e., PCHMA or PMMA) and the desired amount of LR to get concentrations in the range of 0.2–1.8 wt% on 50 mm × 50 mm × 3 mm cleaned glass (BOROFLOAT^®^ Window, Edmund Optics Ltd., York, UK). CHCl_3_ evaporation was performed on a warm plate (30 °C) and in a closed environment. The film thickness was measured to be 23 ± 6, 31 ± 7, and 40 ± 8 µm, respectively, using a Starrett micrometer (The L.S. Starrett Company, Athol, MA, USA). For spectroscopic and microscopic characterization, the polymer films were removed after immersion in water and stored in a desiccator.

### 2.3. Spectroscopic Characterization of Films

UV–VIS absorption measurements were performed using a Cary 5000 spectrometer (Agilent, Santa Clara, CA, USA). Fluorescence spectra in the solid state were measured at room temperature with a Fluorolog^®^-3 spectrofluorometer (Horiba Jobin–Yvon, Horiba Italy, Rome, Italy) equipped with a 450 W xenon arc lamp and double-grating both excitation and emission monochromators. The emission quantum yields of the solid samples were obtained by means of a 152 mm diameter “Quanta-φ” integrating sphere, coated with Spectralon^®^, using as excitation source the 450 W xenon lamp coupled with a double-grating monochromator for selecting wavelengths. The Quanta-φ apparatus was coupled to the spectrofluorometer by a 1.5 m fiber-optic bundle in a slit-round configuration, 180 fibers; slit-end termination is 10 mm O.D. × 50 mm long; round-end termination is FR-274; and the sheath is PVC monocoil. The final quantum yields are an average of three distinct measurements carried out on different films sampling areas. Epifluorescence microscopy images were taken by a LED epifluorescence microscope (Schaefer South-East Europe Srl, Rovigo, Italy) equipped with a LED blue and green 5 W epifluorescence illumination and a DeltaPix Invenio 2EIII microscope camera (DeltaPix, Smorum, Denmark).

### 2.4. Optical Efficiency Measurements of LSCs

A home-built equipment setup was utilized to measure the efficiency of the LSCs. Each fluorophore concentration was tested in triplicate. A sample holder with the photovoltaic (PV) module (IXYS SLMD121H08L mono solar cell 86 mm × 14 mm: Voc = 5.04 V, Isc = 50.0 mA, FF > 70%, ηPV = 22%) is placed 2.5 cm above a scattering layer. The PV cell is masked with black tape to match LSC edge (50 mm × 3 mm) so that limiting the stray light to negligible levels. Silicon was used to grease the LSC edge. The other three edges of the LSC were covered with a reflective aluminum tape. An LCS-100 solar simulator (ORIEL, Newport, Stratford, CT, USA) was housed 27.5 cm above the sample. The PV module was connected to a digital potentiometer (AD5242) controlled via I2C by an Arduino Uno (https://www.arduino.cc) microcontroller using I2C master library. A digital multimeter KEITHLEY 2010 (Keithley Instruments, Cleveland, OH, USA) was connected in series with the circuit, between the PV module and the potentiometer, to collect the current as a function of the external load. Conversely, the voltage was measured by connecting the multimeter in parallel to the digital potentiometer. Arduino Uno controlled the multimeter via SCPI language over RS-232 bus using a TTL to RS-232 converter chip (MAX232). Arduino Uno (Arduino LLC, Somerville, MA, USA) was connected to PC via USB port and controlled by a Python script. The measurement cycle began with a signal from PC to Arduino, which set the multimeter parameter to measure current. Then, Arduino began the measure loop, i.e., (1) set the potentiometer to a given value, (2) send a trigger signal to the multimeter, (3) read the measured data, and (4) send the data back to PC. The loop is repeated 256 times for potentiometer values ranging 60 Ω to 1 MΩ. Arduino set the multimeter to measure voltage, and for each potentiometer value, the system recorded 8 data samples, which were subsequently processed by the Python script. A white back scattering layer (Microcellular MCPET reflective sheet, ERGA TAPES Srl, Milan, Italy) was placed beneath the LSC with an air gap of about 5 mm, during the measurements of the current of the PV cell attached to the LSC edges under illumination (I_LSC_). The optical efficiency was reported as η_opt_.

## 3. Results and Discussion

PCHMA was chosen as an alternative host matrix to PMMA due to a low polarity of the cyclohexyl group that would favor the fluorophore dispersibility in the polymer matrix [48]. As PMMA, PCHMA is an amorphous polymer with a high glass-transition temperature of 104 °C, a good transparency, and desirable mechanical properties that make it an appealing polymer matrix for LSC. Perylene-based luminophores are organic π-conjugated molecules well known for their wide absorption, emission efficiency, and light stability. Among these, LR is the most utilized organic dye for LSC applications, thanks to its unique spectroscopic properties and commercial availability [49,50]. We prepared PCHMA and PMMA thin films of several thicknesses (23 ± 6, 31 ± 7, and 40 ± 8 µm) containing different amounts of LR (0.2–1.8 wt%) by drop casting from chloroform solutions. We then determined the performances of the LR/PCHMA and LR/PMMA thin films LSCs using a Si-based PV cell attached to one edge of the solar collector.

The concentration factor (C) of a LSC device is measured by computing the ratio between the maximal current of the PV cell attached to the LSC edges under illumination of a light source (I_LSC_) and the maximal current of the bare cell put directly exposed to the same light source (I_SC_). Such value depends on the geometrical factor (G) of the device, that is the ratio between the area exposed to the source and the collecting area exposed to the PV cell (in our setup G = 16.6), and the optical efficiency (η_opt_), which represents the intrinsic concentration efficiency of a particular host-dye system and, as such, is a fundamental parameter to evaluate its performances. η_opt_ can, therefore, be measured as:

(1) $$\eta_{\mathrm{opt}}\;=\;\frac{C}{G}\;=\;\frac{I_{LSC}}{G\;\times \;I_{SC}}.$$

**Figure 3:**
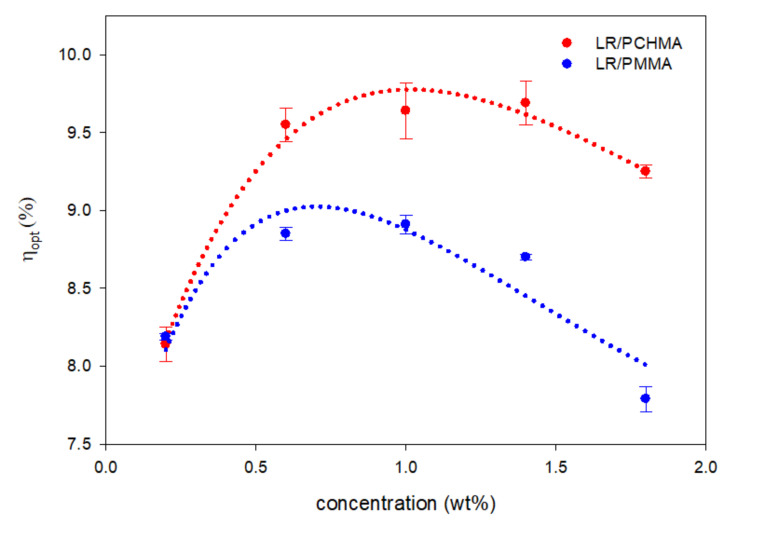
Optical efficiency (η_opt_) % variation of LR/polymer thin films with different dye content (wt%), with a thickness of 23 ± 6 µm and deposited on a 50 mm × 50 mm × 3 mm optically pure glass substrate. The curve was fitted with Equation (2) and the parameters are listed in Table 1. Error bars represent standard deviation in each η_opt_ value (*n* = 3).

As defined, the parameter η_opt_ can be understood in terms of the efficiency of the different phenomena that take place between the photons hitting the LSC surface and the absorption of the emitted ones by the PV cell, i.e., the fraction of photons flux that can arrive at the collection point (minus entropic losses). Measured values of η_opt_ vs. dye concentration for the LR/PCHMA and LR/PMMA systems are shown in Figure 3.

Notably, we observed an increase in the optical efficiency as LR concentration increased in both matrices, up to a concentration of 0.70–1.00 wt%. Beyond that value, η_opt_ decreased. This trend is common in LSC characterization and can be addressed by two counteracting effects caused by the growing content of the fluorophore in the thin films: the light-harvesting efficiency rises as an increased amount of dye molecules in the LSC system can absorb a larger fraction of light; the probability of reabsorption of photons within the LSC film increases because of the larger overlap between absorption and emission spectra, while the fluorescence quantum yield diminishes [51]. In addition, the increased concentration may lead to the formation of less emissive supramolecular aggregates (aggregation-induced quenching phenomena) [52,53]. These processes adversely affect the optical efficiency and consequently the performance of LSC devices. As a result, LR in PCHMA displayed maximum η_opt_ values of about 9.75%, i.e., more than 0.5% higher than those collected from the LSC based on LR/PMMA thin films. It is also worth noting that, compared to PCHMA, we observed the η_opt_ vs. LR concentration trend in LR/PMMA to decrease faster after the maximum [54,55], indicating a larger contribution from optical losses (see Supporting Information).

A common way to investigate η_opt_ is to use the approach of Goetzberger and Greube [13] (Equation (2)) and analyze the different mechanisms that contribute to it separately:

η_opt_ = η_T_ × η_TIR_ × η_QE_ × η_Stokes_ × η_LH_ × η_SA_ × η_host_, (2)

where the different terms represent, respectively, the fraction of energy that is not lost by reflectance of the polymer surface, the efficiency of total internal reflection, fluorescence quantum yield, entropic degradation due to the Stokes shift, absorption spectral mismatch, spectral overlap between emission and absorption, and defects present in the polymer matrix (i.e., scattering defects, impurities, and defects at the interfaces). For a more exhaustive discussion, refer to the Supporting Information file.

Starting from Equation (2), we showed in an earlier work that for LSC devices of given thickness, the value of η_opt_ for a specific host–dye couple is dependent on the dye concentration according to the equation:

η_opt_ = ε^’^ × c × e^−µ^_opt_^× c^ + D, (3)

where c is the concentration of the dye, D is an empirical constant describing the trapped light due to scattering phenomena from surface and bulk defects, and ε′ and μ_opt_ are two empirical constants described as:

(4) $$\varepsilon^{\prime }\;\propto \;h\times e^{-ῑ}$$

(5) $${µ}_{\mathrm{opt}}\;\propto \;{µ}^{\prime \prime }(\mathrm{QY},p)\;\times \;ῑ,$$

where h is the thickness of the thin film, ῑ is the mean path length of the radiation in the optical system, and μ′′ is a term depending on both QY and the probability of fluorescence reabsorption (p). Both ε′ and μ_opt_ are empirical parameters, which allow the comparison of different LSC systems with the same geometry. This approach is described in detail in the Supporting Information.

Notably, ε′ is a coefficient associated with the absorption features of the fluorophore/polymer system, whereas μ_opt_ integrates all the fluorescence quenching phenomena. In this sense, an efficient fluorophore/polymer LSC system is considered when it exhibits a high ε′ and a small μ_opt_ so that the maximum efficiency is shifted to higher dye concentrations (which translate to improved light harvesting properties). The fitting parameters of the optical efficiency variation with LR concentration in LSC films are shown in Table 1.

The fitting parameters of LR/PCHMA and LR/PMMA films appeared somewhat different and only D values were equivalent, indicating the same contribution of nonfluorescent trapping phenomena for samples with the same geometry. Interestingly, the LR/PCHMA system was characterized by a slightly higher ε′ and a smaller μ_opt_ values, thus suggesting that this system was less affected by dissipation phenomena. One may ascribe these differences in performance to the higher dispersibility of the dye at high concentration, favored by the higher hydrophobicity of the polymer matrix.

**Figure 4:**
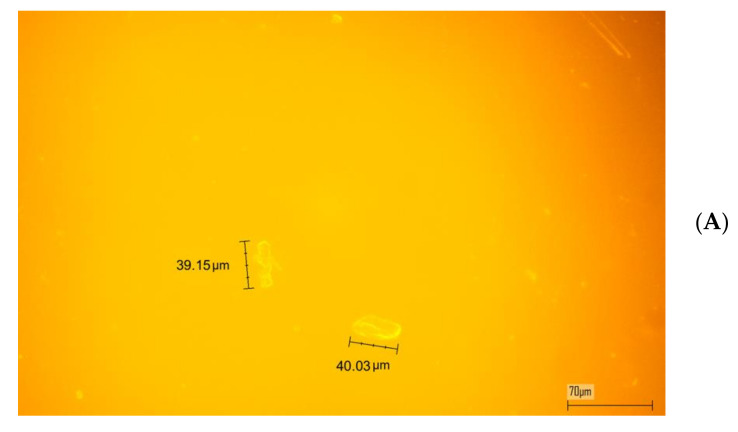

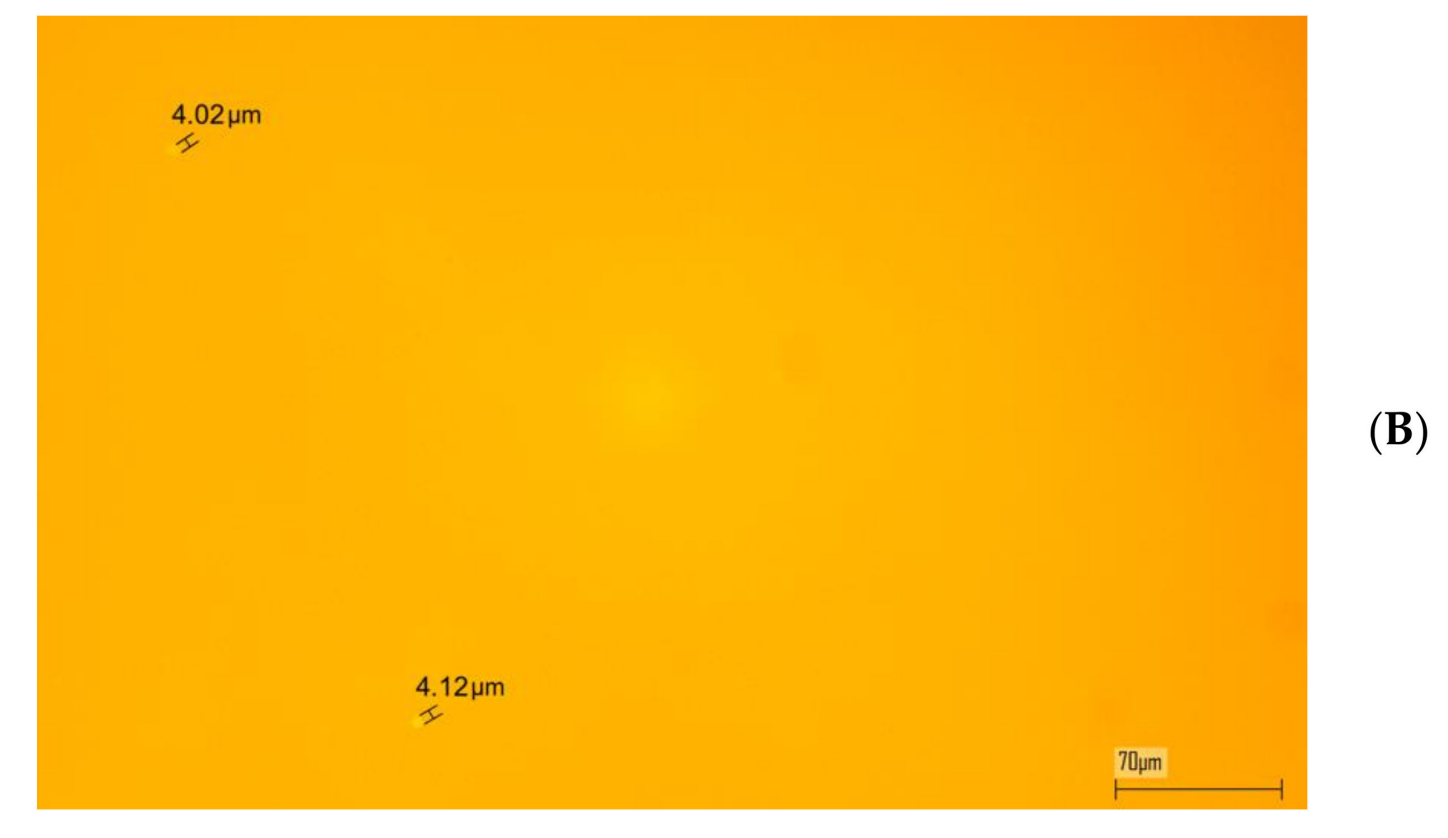
Fluorescent microscopy images of (**A**) poly(methylmethacrylate) (PMMA) and (**B**) PCHMA films doped with 1.4 wt% of LR. Scale bar = 70 μm.

The very high compatibility between LR and the PCHMA was demonstrated by examining the film containing the 1.4 wt% of the fluorophore by epifluorescence microscopy (Figure 4). It was worth noting that negligible microphase separation was observed in the case of the PCHMA film and with the presence a few microsized fluorophore aggregates with average size one order of magnitude lower than the corresponding film based on PMMA.

**Figure 5:**
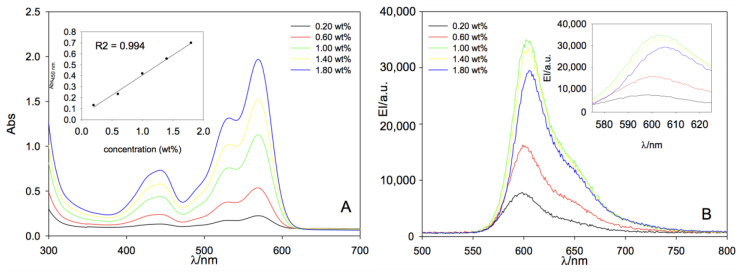
(**A**) UV–VIS absorption. In the inset, the absorbance maximum at 450 nm as a function of LR concentration and (**B**) fluorescence emission spectra PCHMA films at increasing dye concentration. λ_exc_ = 450 nm and film thickness of 23 ± 6 µm.

**Figure 6:**
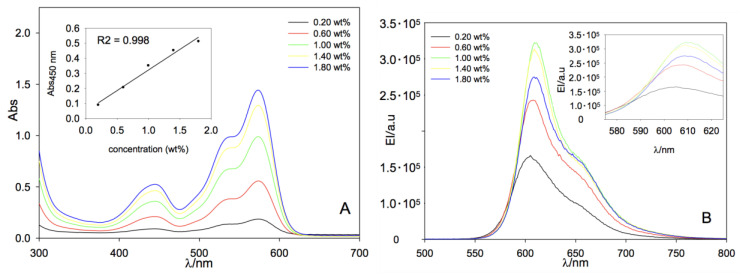
(**A**) UV–VIS absorption. In the inset, the absorbance maximum at 450 nm as a function of LR concentration and (**B**) fluorescence emission spectra PMMA films at increasing dye concentration. λ_exc_ = 450 nm and film thickness of 23 ± 6 µm.

In order to corroborate the optical performance of the aforementioned LR/polymer systems as LSCs, we performed UV–VIS absorption and fluorescence experiments. The optical features of LR embedded in PCHMA are shown in Figure 5 with maximum absorption and emission bands centered at 570 and 604 nm, respectively, with a Stokes shift of 34 nm. LR also absorbs light down to 440 nm with a second structureless band centered at 530 nm. Very similar optical behavior was recorded for LR dispersed in PMMA films with maximum absorption and emission bands found at 573 and 609 nm, respectively, with a Stokes shift of 36 nm [54]. The more polar character of the PMMA matrix was possibly responsible of the 3–5 nm red-shift of the peak maxima. Notably, absorbance increased linearly in PCHMA with the dye concentration as shown in Figure 5A. However, the fluorescence emission intensity increased up to a fluorophore concentration of 1.00 wt%, above which a sharp decrease is observed (Figure 5B), and it is in agreement with the efficiency losses reported in Figure 3. A similar behavior in PMMA was observed in Figure 6 with both absorption and emission trends confirmed with LR content.

Nevertheless, by comparing the absorption intensities, LR in PCHMA displayed higher absorbance values than the corresponding samples in PMMA, reaching a maximum of about 2 and 1.5 respectively, at their maximum content of 1.8 wt%. These data support the higher solar harvesting characteristics of LR in PCHMA, which translate to better η_opt_ values.

Looking at the emission bands, the insets in Figure 5B and Figure 6B evidenced a bathochromic shift of the maximum peak fluorescence of about 6 and 8 nm for LR/PCHMA and LR/PMMA systems, respectively. Such phenomenon can be addressed to a combination of effects: (i) dye molecules could aggregate and exhibit a red-shift in the emission spectra [56] and (ii) auto-absorption phenomena (inner filter effect) could occur because of spectral overlap. This last seemed more evident for the PMMA films.

**Figure 7:**
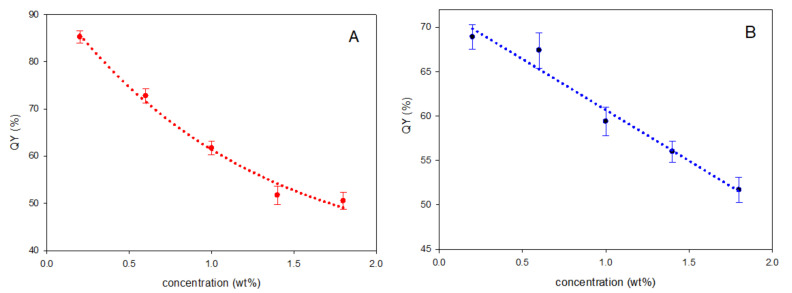
Quantum yields (QY) (%) of LR in (**A**) PCHMA and (**B**) PMMA films versus fluorophore concentration (wt%) with a thickness of 23 ± 6 µm. Error bars represent standard deviation in each QY value (*n* = 3).

In Figure 7, we report the measured quantum yields (QY) as a function of LR concentration for both PCHMA and PMMA. We observed a constant and linear decrease in the QY values of up to 50% in PMMA (Figure 5B), which in agreement with what is reported in the literature [54]. Conversely, LR in PCHMA (Figure 5A) showed higher QY values for fluorophore contents <1 wt%, which tended to level off to a plateau of 50% at the higher concentrations. Although, there were no substantial differences between the absolute QY values of LR in PCHMA and in PMMA, considering also a typical deviation of 10% of each quantum yield measurement, emission concentration appeared more adversely affected in PMMA. This could be attributed to the presence of larger emissive LR aggregates in PMMA (Figure 4) that possibly worsen the fluorescence collection at the edge of the collector.

Future experiments will be devoted to time-resolved fluorescence that could eventually provide useful information to dissect the relative contributions from quenching and reabsorption, and the presence of possible dynamic processes attributed to aggregates within the films [57].

**Figure 8:**
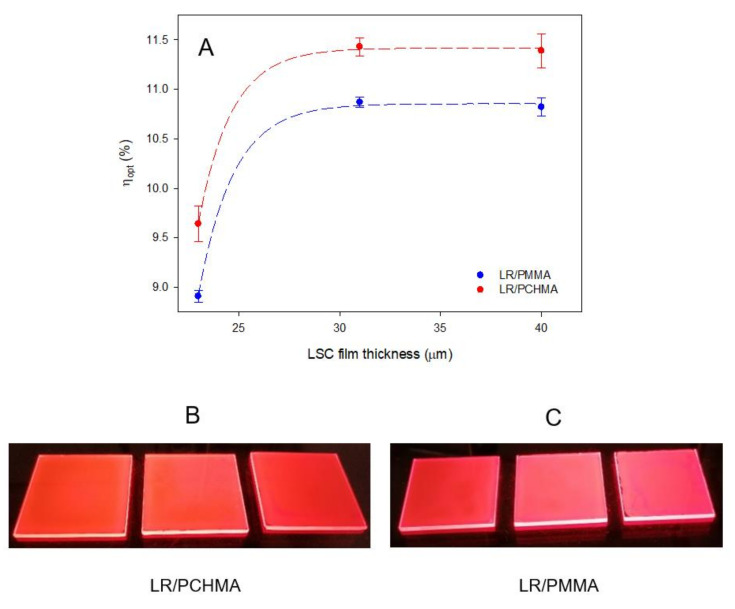
(**A**) η_opt_ of LSC systems versus LSC film thickness at 1.00 wt% of LR for both polymeric matrices investigated and (**B**,**C**) pictures of the same LR/polymer LSC (50 mm × 50 mm × 3 mm) under the excitation with a Dark Reader 46B transilluminator (∼450 nm) with increasing film thickness (from left to right). Error bars represent standard deviation in each η_opt_ value (*n* = 3).

Finally, we evaluated the influence of the film thickness on η_opt_, aimed to determine the best working conditions for an LSC device. To do so, we varied the amount of polymer during the fabrication to produce LSC systems with film thicknesses of 23 ± 6, 31 ± 7, and 40 ± 8 µm and a fixed LR concentration of 1.00 wt%. The results are shown in Figure 8.

**Figure 9:**
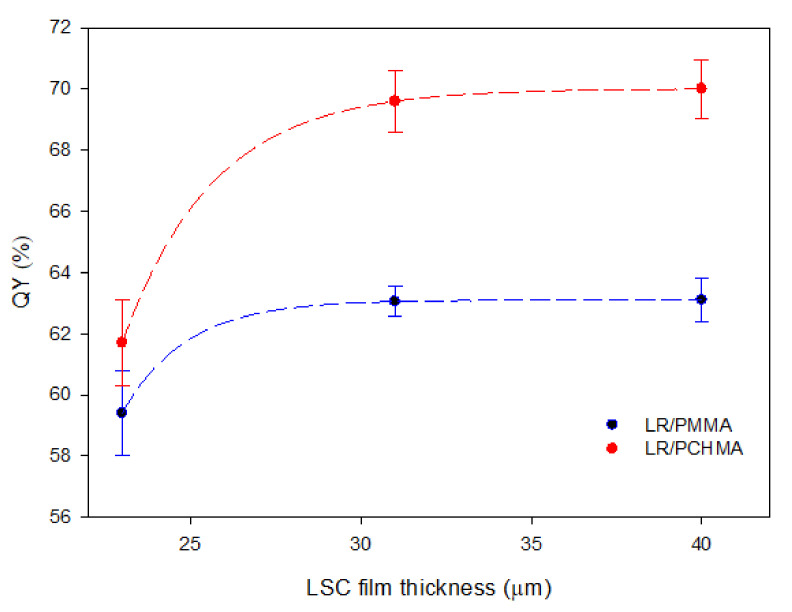
Quantum yield (%) of LR/polymer thin films as a function of LSC film thickness at 1.00 wt% of LR for both polymeric matrices studied. Error bars represent standard deviation in each QY value (*n* = 3).

It is worth noting that all LR/PCHMA systems showed higher η_opt_ values when compared to PMMA-based LSCs, thus confirming the ability of the PCHMA matrix in the effective dispersion of LR fluorophores. Maximum optical efficiencies of about 11.4% and 10.7% were obtained for 31 ± 7 µm thick LR/PCHMA and LR/PMMA LSCs, respectively. The surface texture appeared also homogeneous without the typical roughness conferred by the chromophoric clusters (Figure 8B,C). This explanation is eventually supported by the determination of the QY values of the same LSC systems. As shown in Figure 9, after an initial growing of QY, a plateau was reached and corresponding to the 70% in PCHMA and 64% in PMMA, respectively, which is in strong agreement with the η_opt_ data.

## 4. Conclusions

This work examines the potential offered by the replacement of the PMMA matrix typically employed in LSC systems with a less polar one such as PCHMA. LSCs prepared from thin film of PCHMA and containing different amounts of LR (i.e., from 0.2 to 1.8 wt%) showed η_opt_ values higher than those measured from the corresponding PMMA films. It was worth noting that LSCs prepared with 1.00 wt% LR in PCHMA reached maximum η_opt_ of 11.4%, i.e., about 0.7% higher than equivalent devices prepared with PMMA. This increment in performances was attributed to a more effective dispersion of the perylene-based fluorophore in the less polar polyacrylate matrix, which resulted in higher solar harvesting characteristics, reduced influence of dissipative phenomena, and higher quantum efficiency. These observations support the use of PCHMA in the preparation of high-performance LSCs and incentivizes the investigation of similar less polar matrices for these applications in order to better match the chemical properties of the dyes employed.

## Figures and Tables

**Table 1:** Fitting parameters of the optical efficiency data measured for Lumogen F Red 305 (LR)/poly(cyclohexylmethacrylate) (PCHMA) and LR/poly(methylmethacrylate) (PMMA) films with a thickness of 23 ± 6 µm.

Entry	ε^’^	µ_opt_	D
LR/PCHMA	8.5 ± 0.9	0.98 ± 0.05	6.9 ± 0.3
LR/PMMA	7.7 ± 1.1	1.42 ± 0.16	6.9 ± 0.5
